# Subsequent Primary Neoplasms and Mortality Among Survivors of Childhood Cancer in Alberta, Canada

**DOI:** 10.3390/cancers18040694

**Published:** 2026-02-20

**Authors:** King Wa Tam, Tona M. Pitt, Kathleen Reynolds, Maria Spavor, Tony H. Truong, Jennifer Giles, Gregory M. T. Guilcher, Natalie Logie, Iqra Rahamatullah, Fiona Schulte, Miranda M. Fidler-Benaoudia

**Affiliations:** 1 Department of Oncology, University of Calgary, 2500 University Drive NW, Calgary, AB T2N 1N4, Canada; kingwa.tam@ucalgary.ca (K.W.T.); tpitt@ucalgary.ca (T.M.P.); tony.truong@albertahealthservices.ca (T.H.T.); greg.guilcher@albertahealthservices.ca (G.M.T.G.); natalie.logie@albertahealthservices.ca (N.L.); fiona.schulte@ucalgary.ca (F.S.); 2 Department of Paediatrics, University of Calgary, 2500 University Drive NW, Calgary, AB T2N 1N4, Canada; 3 Department of Cancer Epidemiology and Prevention Research, Cancer Care Alberta, Arthur Child Comprehensive Cancer Centre, 3395 Hospital Drive NW, Calgary, AB T2N 5G2, Canada; 4 Department of Family Medicine, Cumming School of Medicine, University of Calgary, 2500 University Drive NW, Calgary, AB T2N 1N4, Canada; kathya.reynolds@albertahealthservices.ca; 5 Long Term Survivors Clinic, Alberta Children’s Hospital, 28 Oki Drive NW, Calgary, AB T3B 6A8, Canada; jennifer.giles@albertahealthservices.ca; 6 Department of Paediatrics, University of Alberta, 11405-87 Avenue NW, Edmonton, AB T6G 1C9, Canada; maria.spavor@albertahealthservices.ca; 7 Northern Alberta Childhood Cancer Survivor Program, 11135-84 Avenue NW, Edmonton, AB T6G 0V9, Canada; 8 Haematology, Oncology, and Transplant Program, Alberta Children’s Hospital, 28 Oki Drive NW, Calgary, AB T3B 6A8, Canada; 9 Department of Community Health Sciences, University of Calgary, 2500 University Drive NW, Calgary, AB T2N 1N4, Canada

**Keywords:** pediatric oncology, epidemiology, late effects, long-term survival, subsequent primary neoplasm

## Abstract

**Background**: While research shows childhood cancer survivors experience elevated subsequent primary neoplasm (SPN) and premature mortality risks, few studies have included contemporary survivors. **Methods**: This study quantifies the risk of SPNs and mortality among modern survivors of childhood cancer. Utilizing a retrospective, population-based cohort of individuals diagnosed with cancer before the age of 18 in Alberta, Canada (2001–2018), we evaluated their risks of SPNs and mortality compared to the general population in Alberta, overall and after 5-year survival, using standardized mortality and incidence ratios, and absolute excess risks per 10,000 person-years. **Results**: Among 2581 survivors, including 1385 5-year survivors, 50 individuals developed at least one SPNs and 408 deaths were observed, with 21 SPNs and 38 deaths occurring after 5-year survival. The SPN incidence was 13.3- (95% CI: 9.8–17.5) and 10.0-fold (95% CI: 6.2–15.2) higher than expected overall and in 5-year survivors, respectively, with risks varying depending on the treatment received. For mortality, survivors experienced 62.5-fold (95% CI: 56.5–68.8) higher mortality than expected overall, equating to 233.9 (95% CI: 210.8–257.0) excess deaths per 10,000 person-years, with corresponding risks among 5-year survivors at 10.9 (95% CI: 7.7–15.0) and 43.8 (95% CI: 28.4–59.1), respectively. The excess deaths were predominantly due to recurrence/progression (89.9% overall, 66.4% in 5-year survivors), with SPNs and non-neoplastic causes contributing more excess deaths with increasing follow-up time. Risks for mortality included treatment and cancer type. **Conclusions**: Contemporary childhood cancer survivors in Alberta experience substantial excess SPNs and mortality, highlighting the need for long-term surveillance and tailored risk mitigation interventions.

## Simple Summary

Children diagnosed with cancer face a higher risk of chronic health conditions and death both in the short term and long term. With improvements in treatment over the last 20 years, the risk of late effects and overall survival have changed, but there are relatively few studies examining how these risks changed over time. This study followed 2581 children diagnosed from 2001 to 2018 in Alberta, Canada, until 31 December 2018. We identified those who developed a subsquent cancer and those who died during our follow-up and compared these rates to what was expected in the general population of Alberta. Children diagnosed with cancer experienced more subsequent cancers and deaths compared to the general population; these poor outcomes differed by initial cancer type and the treatment received, and highlight the need for long-term follow-up care for survivors of childhood cancer.

## 1. Introduction

Survivorship rates for childhood cancer have improved dramatically since the 1970s, with 5-year survival increasing from approximately 58% to over 80% for children diagnosed in developed countries [1,2,3]. Despite these encouraging statistics, survivors of childhood cancer face significant long-term health challenges due to their cancer and its treatment that warrant further investigation [4,5]. Research indicates that childhood cancer survivors experience heightened risks of an array of adverse outcomes [6,7,8,9,10,11,12,13], with the most prominent late effects being subsequent primary neoplasms (SPNs) [14,15,16,17] and premature mortality [18,19,20,21,22,23]. Previous reports on SPNs have documented risks beginning at diagnosis and after 5-year survival to understand the burden overall and specifically among long-term survivors [24,25,26,27], while mortality studies from the United States, Great Britain, Australia, and Europe have investigated the risk of death up to 5-year survival as a benchmark for cure [19,23,28,29,30], as well as after 5-year survival in order to understand increases in late mortality [4,5,31]. While these historical investigations significantly advanced our understanding on the impact of a childhood cancer diagnosis and its treatment on late effects and survival, treatment modalities and supportive care strategies have changed substantially in the last decades in an effort to achieve cure while reducing the likelihood of late effects, bringing into question whether historical findings are generalizable to contemporary survivors of childhood cancer [32].

Indeed, since the early 2000s, more precise diagnostics, such as molecular biological markers for better risk stratification and advances in imaging techniques for better staging and disease response evaluations, have led to improved outcomes and a reduction in late effects [33,34,35,36]. Advances in childhood cancer treatments have also occurred, including the use of immunotherapy and targeted therapies, particularly for hematological malignancies [37,38,39,40,41,42,43,44]. Radiotherapy techniques have also moved towards volumetric planning and decreases in clinical target volume and planning target volume margins, while the introduction of conformal radiotherapy techniques, such as intensity-modulated radiotherapy, may reduce high-dose radiotherapy regions outside the target region, and proton therapy may reduce the integral dose [45]. Finally, developments in survivorship have enabled clinicians to better prevent, detect, and address late effects through supportive care measures, such as the use of cardioprotective and evidence-informed screening strategies [41]. When combined, these efforts have led to more childhood cancer patients being cured, with the expectation being that they will also have a better quality of life as a survivor. However, long-term follow-up is needed to understand whether these initiatives led to meaningful improvements as anticipated [4,5,41,45].

Thus, the primary objective of this study is to quantify the excess risk of SPNs and mortality among contemporary survivors of childhood cancer in Alberta, Canada, compared to that expected in the general population, both overall and after 5-year survival. The secondary objective is to identify explanatory demographic and clinical factors that contribute to these excess risks. The findings from this study will not only fill an existing gap in the literature, but may also inform initiatives aimed at reducing morbidity and mortality in this population through preventive interventions and tailored follow-up care.

## 2. Methods

### 2.1. Study Design and Participants

This study is part of the Alberta Childhood Cancer Survivorship Research Program, which has been described in detail previously [46]. Briefly, this study utilized a retrospective, population-based cohort of Alberta residents diagnosed with a first primary neoplasm between the ages of 0 and 17 years, from 1 January 2001 to 31 December 2018. The term “survivor” was defined according to the National Cancer Institute definition where “an individual is considered a cancer survivor from the time of diagnosis through the balance of life,” regardless of whether the individual is living free from disease or living with cancer [47]. The cohort was established using data from the Alberta Cancer Registry, with all cancer diagnoses meeting the International Classification of Childhood Cancer, third revision (ICCC-3) included [48]. The treatment information was obtained through a linkage with the Cancer in Young People in Canada (CYP-C) consortium for 1745 survivors, with a manual chart review being conducted for the remaining survivors using the same data dictionary as the CYP-C initiative [49]. Ethical approval was obtained from the Health Research Ethics Board of Alberta (HREBA.CC-20-0110), with a consent waiver granted due to the study’s retrospective nature and minimal risk to participants.

### 2.2. SPN and Death Ascertainment

SPN data were obtained through linkage with the Alberta Cancer Registry, where all subsequent invasive cancers and in situ bladder cancers meeting the Surveillance, Epidemiology and End Results Program (SEER)’s Solid Tumor Rules and SEER’s Multiple Primary Rules were identified [50,51]; accordingly, disease recurrence or progression was not considered as new primary cancer. SPNs were coded with ICD-O codes and grouped into the following larger organ systems to match provincial cancer definitions for general population cancer rates: head and neck, liver and intrahepatic bile ducts, bone and connective tissues, breast, ovary, kidney, central nervous system (CNS), endocrine glands, non-Hodgkin lymphoma, Hodgkin lymphoma, leukemia, other blood, and other ill-defined and unknown [52]. SPN risk was evaluated both overall and by SPN type, with only the first SPN of interest included in the analysis. 

The dates and causes of death were obtained from the Alberta Cancer Registry via an ongoing linkage to the provincial Vital Statistics database. The underlying cause of death, as listed on the death certificate using the International Classification of Diseases, tenth edition (ICD-10) codes, was categorized according to the ICD groupings used by the Albertan government to allow for comparison with the general population’s mortality rates [53]. To determine whether a cancer-related death was due to the initial primary neoplasm or an SPN, the death ICD-10 code was compared with the ICD-Oncology (ICD-O) topography code for each diagnosis. For non-concordant cases, medical charts were reviewed to identify the appropriate cause of death; deaths were conservatively classified as recurrence/progression of the childhood cancer if the death could not be conclusively attributed to a specific cancer diagnosis (n = 2). 

### 2.3. Statistical Analysis

Follow-up began at diagnosis or 5-year survival and continued until the first instance of emigration from Alberta, death, or the study end date (31 December 2018), with the date of the first SPN of interest also being a censoring point for analyses where SPNs were the outcome of interest; emigration was determined using registration dates for the Alberta Health Care Insurance Plan, which provides universal access to healthcare services for all Albertan residents. For SPNs, cumulative incidence as a function of follow-up time was calculated with death as a competing risk. Similarly, cumulative mortality probabilities for all-cause and cause-specific mortality were calculated as a function of follow-up time, where causes of death other than the one of interest were considered as competing risks.

Standard cohort techniques were used to calculate absolute excess risks (AERs), standardized incidence ratios (SIRs), and standardized mortality ratios (SMRs) [54]. The AER, which is the mean excess number of cancers (for SPN analyses) or deaths (for mortality analyses) per 10,000 survivors per year, was calculated by subtracting the expected number of events from the observed number of events in the general population of Alberta, dividing by person-years at risk and then multiplying by 10,000. SIRs and SMRs, which provide the multiplicative excess in cancers and deaths, respectively, were calculated by dividing the observed number of events by the expected number. The expected number of cancers and deaths were derived by multiplying the number of person-years accrued, stratified by sex, attained age (5-year bands) and calendar year (1-year bands), by the corresponding rate for the general population of Alberta and summing appropriately. The general population rates for cancer and mortality were obtained from the Aberta Cancer Registry and Government of Alberta, respectively [55]. AERs, SIRs, and SMRs were stratified by covariates, including sex, age at diagnosis, regional health zone at diagnosis, and ICCC-3 diagnosis, with likelihood ratio tests used to assess heterogeneity or trends, with two-sided *p*-values less than 0.05 considered statistically significant. All the statistical analyses were performed using Stata 18.0.

## 3. Results

**Figure 1:**
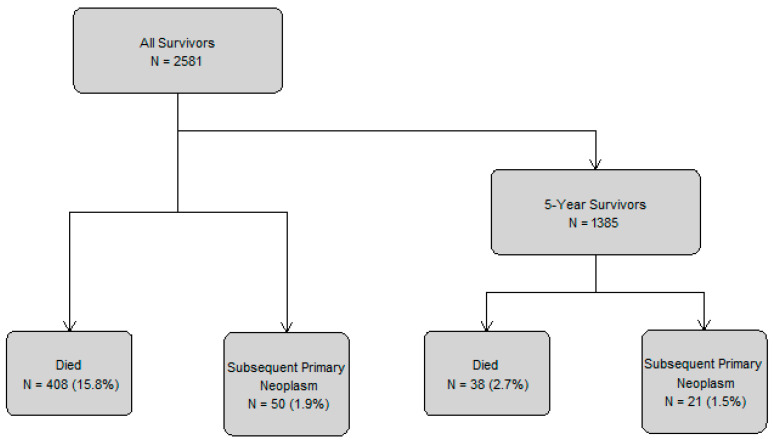
Flowchart of childhood cancer survivors and outcomes.

The study cohort included 2581 survivors of childhood cancer, of whom 1385 were 5-year survivors (Figure 1 and Table 1). Overall, the majority of the cohort was male (52.5%), the median age at diagnosis was 7.6 years (IQR: 2.9–14.1), and survivors primarily resided in Calgary (35.5%) or Edmonton (31.6%). The most common diagnoses were leukemias (25.3%), CNS tumors (24.2%), and lymphomas (14.9%). Most survivors received chemotherapy (68.1%), nearly half underwent surgery (44.1%) and around one third received radiotherapy (31.3%). At the study exit, 17 164 person-years of follow-up were accrued and the median follow-up time from diagnosis was 5.6 years (IQR: 1.9–10.9). Among the 5-year survivors, characteristics were generally similar, though 7890 person-years of follow-up were accrued and themedian follow-up time was longer at 10.3 years (IQR: 7.5–13.7).

### 3.1. Survivors Overall

Overall, 50 (1.9%) survivors developed at least one SPN, which was 13.3 times (95% CI: 9.8–17.5) higher than expected, equating to 27.1 (95% CI: 19.0–35.3) excess neoplasms (Table 2). SPNs were most frequently hematologic, CNS tumors or bone/connective tissue cancers, with the largest AERs observed for subsequent leukemias (AER: 6.1; 95% CI: 2.3–9.9) and bone and connective tissue tumors (AER: 5.1; 95% CI: 1.6–8.5).

The risk factors for developing an SPN among survivors overall included receiving chemotherapy, radiotherapy, or a stem cell transplant as treatment, with multiplicative (SIR: 61.3; 95% CI: 35.0–99.5) and additive (AER: 133.9; 95% CI: 67.2–200.7) risks being the greatest for stem cell transplant recipients (Table 3). Survivors of leukemia, lymphoma, and soft tissue tumors were statistically significantly more at risk than the general population, with 30.9, 48.9, and 54.5 excess neoplasms per 10,000 person-years observed, respectively. 

**Figure 2:**
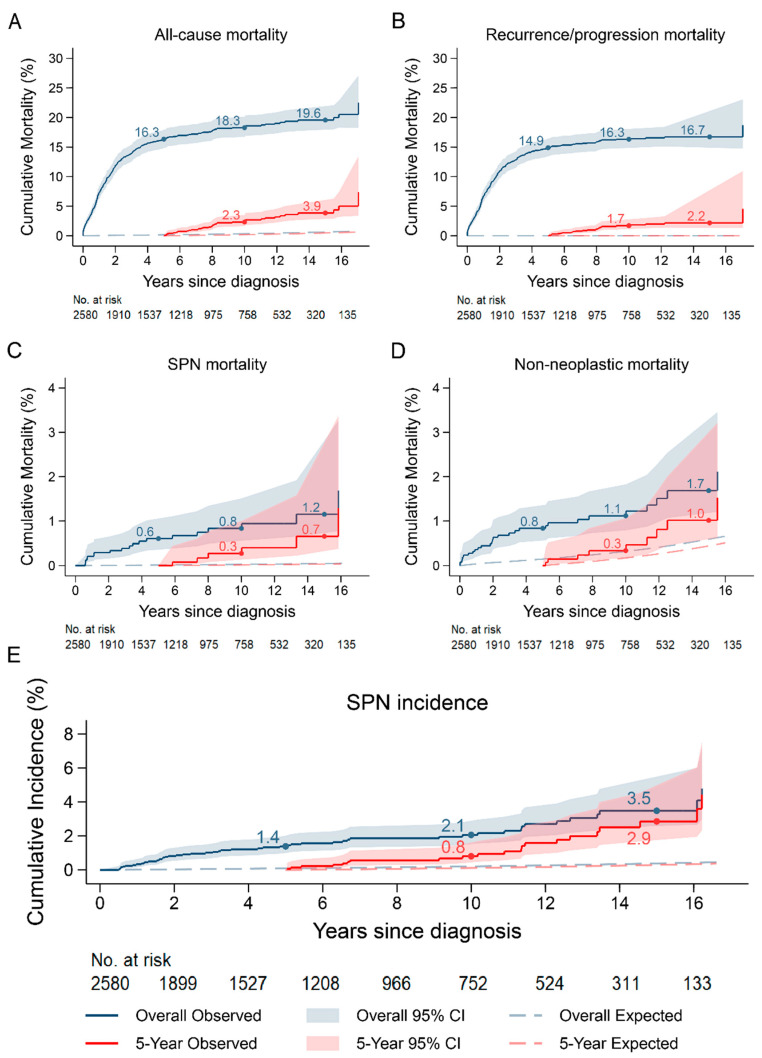
(**A**) Cumulative all-cause mortality in overall and 5-year childhood cancer survivors. (**B**) Cumulative recurrence/progression mortality in overall and 5-year childhood cancer survivors. (**C**) Cumulative SPN mortality in overall and 5-year childhood cancer survivors. (**D**) Cumulative non-neoplastic mortality in overall and 5-year childhood cancer survivors. (**E**) Cumulative SPN incidence in childhood cancer survivors overall and after 5-year survival.

The cumulative SPN incidence increased steadily over time, ultimately reaching 3.5% (95% CI: 2.5–4.8) at 15 years post diagnosis, which was higher than the 0.4% that was expected (Figure 2E).

Among the 2581 survivors, 408 deaths (15.8%) were observed, of which 361 (88.5%) were due to recurrence/progression, 19 (4.7%) were due to SPNs, and 28 (6.9%) were due to non-neoplastic causes (Table 4). The overall cohort experienced 62.5-times (95% CI: 56.5–68.8) more deaths than expected, corresponding to 233.9 (95% CI: 210.8–257.0) excess deaths per 10,000 person-years. Of these excess deaths, 94.5% were attributable to cancer in the overall cohort (AER: 221.1; 95% CI: 198.8–243.4); this was largely due to recurrence/progression deaths (89.9% of total AER), though SPN-related mortality was also significantly elevated (SMR: 37.9; 95% CI: 22.8–59.2), corresponding to an AER of 10.8 (4.6% of total AER). Mortality from non-neoplastic causes was also elevated (SMR: 4.6; 95% CI: 3.1–6.7), contributing a further 12.8 excess deaths (5.5% of total AER); two thirds of the excess deaths were due to health-related causes (SMR: 9.3, 95% CI: 5.4–14.8; AER: 8.8, 95% CI: 4.1–13.5) and one third of the excess deaths were due to external causes (SMR: 2.6, 95% CI: 1.3–4.7; AER: 4.0, 95% CI: 0.2–7.8), the majority of which were due to suicide though this was not statistically significantly elevated compared to the general population (SMR: 4.0, 95% CI: 1.3–9.4; AER: 2.2, 95% CI: −0.4–4.7).

The number of excess deaths due to recurrence/progression significantly varied according to the childhood cancer diagnosis (*p* < 0.001), diagnosis period (*p* < 0.001), follow-up time (*p* < 0.001), and treatment received (all *p* < 0.001) (Table 5 and Table S1). Specifically, all childhood cancer diagnoses experienced statistically significantly elevated risks, except diagnoses of retinoblastoma and other/unspecified tumors; AERs ranged from 68.6 to 572.1 per 10,000 person-years, with the highest AERs observed among the survivors of malignant bone tumors (AER: 572.1; 95% CI: 397.0–747.3). The survivors diagnosed more recently and, complementarily, those with less follow-up time, also had higher AERs for recurrence/progression deaths. By treatment exposures, the survivors treated with stem cell transplant (AER: 511.6; 95% CI: 385.3–637.9), radiotherapy (AER: 370.8; 95% CI: 319.3–422.3), or chemotherapy (AER: 251.9; 95% CI: 223.3–280.6) experienced higher AERs than those who did not receive these therapies, while those treated with surgery had lower absolute excesses. For SPN deaths, receiving stem cell transplant, radiotherapy, or chemotherapy (*p* < 0.001) were the only significant risk factors identified. While statistically significant heterogeneity across childhood cancer diagnoses was not observed for SPN deaths, likely due to insufficient events in most strata, significant absolute excesses in SPN deaths were observed for the survivors of leukemia (AER: 17.2; 95% CI: 5.1–29.2) and lymphoma (AER: 14.1; 95% CI: 0.0–28.3). For non-neoplastic deaths, no risk factors were identified, though survivors of lymphoma were observed to have significant excess deaths (AER: 24.4; 95% CI: 4.4–44.5).

The cumulative mortality in the first 5 years after diagnosis was 16.3% (95% CI: 14.8–17.9) compared with the 0.16% that was expected, after which it plateaued to reach 19.6% at 15 years post diagnosis (Figure 2A). This early rise was driven primarily by recurrence/progression deaths, with a cumulative mortality of 14.9% (95% CI: 13.5–16.4) at 5 years post diagnosis observed (Figure 2B). SPNs and non-neoplastic mortality reached 1.2% (95% CI: 0.6–1.9) and 1.7% (95% CI: 1.1–2.5), respectively, at 15 years post diagnosis, which was significantly higher than expected, at 0.01% and 0.15% respectively (Figure 2C,D).

### 3.2. 5-Year Survivors

There were 21 SPNs observed after 5-year survival; multiplicative (SMR: 10.0; 95% CI: 6.2–15.2) and absolute excesses (AER: 24.1; 95% CI: 12.6–35.5) were comparable to the overall cohort. However, CNS and endocrine tumors contributed the most to the excess number of neoplasms observed. The SPN risk increased with longer follow-up times, with the AER rising from 15.0 (95% CI: 3.7–26.3) during 5–9 years of follow-up to 42.0 (95% CI: 16.2–67.7) at 10+ years (*p* < 0.05) (Table 5). Treatment modalities also influenced SPN risk; while the SIR was highest for 5-year survivors who received a stem cell transplant (28.4; 95% CI: 7.7–72.7), the AER was not statistically significant, whereas survivors treated with radiotherapy or chemotherapy experienced 54.1 (95% CI: 24.1–84.1) and 32.7 (95% CI: 16.9–48.5) excess neoplasms, respectively, per 10,000 person-years. Despite childhood cancer diagnosis again not being identified as a risk factor, survivors of lymphoma remained statistically significantly more at risk than the general population, with 58.2 (95% CI: 15.4–100.9) excess neoplasms per 10,000 person-years. 

The cumulative incidence curve for SPNs was comparable to that of the overall survivors, with the risks at 15 years post diagnosis being 2.9% (95% CI: 1.5–4.0) and significantly in excess of the 0.3% expected (Figure 2E).

There were 38 deaths (2.7%) among the 5-year survivors: 23 (60.5%) were due to recurrence/progression, 6 (15.8%) due to SPNs, and 9 (23.7%) due to non-neoplastic causes (Table 4). SMRs and AERs were 10.9 (95% CI: 7.7–15.0) and 43.8 (95% CI: 28.4–59.1), respectively. Compared with the risks in the overall cohort, the proportion of excess deaths due to recurrence/progression decreased to 66.4%, while SPN- and non-neoplastic-related mortality increased to 16.7% each. External causes accounted for nearly all (88.9%) of the excess non-neoplastic deaths observed.

Elevated recurrence/progression-related mortality persisted for CNS tumors (AER: 45.9; 95% CI: 14.1–77.7) and leukemias (22.8; 95% CI: 2.8–42.7) (Table S2). Similarly to the overall survivors, the 5-year survivors treated with chemotherapy (*p* = 0.042), radiotherapy (*p* < 0.001), or stem cell transplant (*p* < 0.001) experienced higher AERs due to recurrence/progression than those who did not. No other risk factors were identified for recurrence/progression-, SPN- or non-neoplastic-related death among the 5-year survivors.

Cumulative mortality was statistically significantly lower than that of the overall survivors, at 3.9% 15 years post diagnosis, which was driven by the decrease in recurrence/progression mortality given that mortality proportions were comparable for other causes (Figure 2A–D).

## 4. Discussion

In this population-based cohort of 2851 childhood cancer survivors in Alberta, Canada, spanning 2001–2018, we observed significant risks for SPNs and premature mortality compared with the general population. Uniquely, this study estimates the multiplicative and additive excesses of SPNs and death, and assesses the risk factors for each, beginning at diagnosis and 5-year survival; in doing so, this approach considers the immediate risks to all childhood cancer survivors rather than restricting the analysis to 5-year survivors, as is common practice. Our findings underscore the continued need to achieve long-term cure, as recurrence/progression of the childhood cancer remains the main contributor to premature mortality, as well as the critical need for long-term, risk-based follow-up care to address the ongoing health challenges faced by the survivors of childhood cancer upon aging.

The survivors of childhood cancer are known to have a significantly increased risk of developing an SPN, with our findings aligning with studies from North America and the United Kingdom, where risks of 4- to 7-fold higher than expected among 5-year survivors were reported [25,27,56,57,58,59,60], as well as a with study from Ontario where a 9.9-fold risk for overall survivors was found [60]. While the SPN risk estimates in this cohort were higher, with a 13.3-fold increase overall and a 10.0-fold increase in 5-year survivors, the precision of 95% CIs should be considered. Treatment exposures affected the risk of SPNs, and survivors of leukemia, lymphoma, and soft tissue tumors had higher risk of SPNs. These findings correspond with the literature, where chemotherapy individually and jointly with radiotherapy increases the risk of subsequent solid cancers and sarcomas [58,61,62]. Additionally, CNS SPNs, which accounted for 10% of all SPNs in this sample, have been associated with radiotherapy exposure in the treatment of acute lymphoblastic leukemia, lymphoblastic lymphoma, and primary CNS tumors [60,61,63], further aligning with our findings. Genetic predispositions and lifestyle factors also contribute to SPN risk, and although these were unmeasured in this study the inclusion of genetic counseling and education on living healthy should be mandatory in survivorship care [64,65].

Our observed overall and cause-specific mortality risk estimates are largely consistent with the literature [19,23,28,30,31,66,67,68,69,70,71]. Specifically, our 5-year survivor results align locally with a pan-Canadian study (diagnoses from 1992 to 2017) and British Columbia study (diagnoses from 1970 to 1995) where SMRs of 9.4 [67] and 9.1 [69] were reported, respectively. Comparing with European estimates, a study combining data on those diagnosed before the age of 21 years from 11 countries in Europe (diagnoses from 1940 to 2008) indicated an SMR of 9.9 [30], with other cohorts in Great Britain (diagnoses 1940–2006) and Switzerland (diagnoses 1976–2007), demonstrating SMRs of 9.1 and 10.2, respectively [19,68]. The US estimates were similar, including survivors of childhood cancer aged up to 19 years (diagnoses from 1962 to 2012; SMR: 7.6), 20 years (diagnoses from 1974 to 2000; SMR: 8.9), and 21 years (diagnoses from 1970 to 1999; SMR: 5.6) [31,70,71]. Considering contemporary data that are more comparable to our cohort, Byrne et al. reported an SMR of 27.1 (95% CI: 24.0–30.5) for European survivors of childhood cancer diagnosed from 2000 to 2008 [30], and Ehrhardt et al. reported an SMR of 19.3 (95% CI: 16.7–22.2) for American survivors of childhood cancer diagnosed from 2000 to 2012 [70]. While these results are notably higher than those of our study, these most recent era-specific SMRs were calculated from larger, historical cohorts where limited information regarding specific patient characteristics and person-year of follow-up time was provided for the contemporary survivors included; without this information, it is difficult to speculate on the reasons for discordance, though we anticipate that the observed differences may reflect differing follow-up times, included cancer types, and access to treatment [30].

In this study, both relative and absolute mortality risks were observed to decline with increasing time since diagnosis. Others suggest a potential U-shaped pattern in excess mortality, where absolute risk rises again after 20 years due to late effects; thus, reassessment with additional follow-up will be key to assess whether this pattern remains in contemporary survivors [23]. Additionally, the highest mortality risks were found among survivors of malignant bone tumors, soft tissue sarcomas, hepatic tumors, and CNS tumors; previous studies have similarly reported higher mortalities of similar magnitudes for these survivor populations [23]. Recurrence/progression was the most frequently observed cause of death in the 5-year survivors and aligned with previous estimates of the recurrence/progression-related proportion of deaths [30,66] and of AERs [19,31,68]. The treatment received was identified as a risk factor, with survivors who received stem cell transplant, radiotherapy, or chemotherapy experiencing higher excess mortality than those who did not. These treatments have known dose-dependent late effects such as SPNs and cardiovascular diseases, increasing mortality risks [27,72]. Furthermore, the use of a stem cell transplant is often reserved for relapsed/refractory disease or cancers classified as high risk, due to inherent biological features known to make the cancer refractory or likely to recur after standard frontline therapy, and are thus inherently associated with poorer prognosis [73,74,75].

A notable finding in the study was the elevated suicide SMR risk among survivors, with all five suicides in this cohort occurring among young males (detailed results suppressed due to small numbers). Although survivors of childhood cancer are known to face increased risks of suicidal ideation and psychiatric disorders, including depression and anxiety, the evidence on suicide mortality remains mixed. While a meta-analysis conducted among survivors of childhood cancer found the overall excess in suicide deaths not to be statistically significant compared to matched controls and siblings, subgroups such as male survivors and those diagnosed during adolescence appear more vulnerable [76,77]. In Alberta, childhood cancer survivors face additional psychosocial challenges as they transition from pediatric to adult care, including ongoing and continued psychosocial support during and after initial cancer treatment [77]. These findings should thus be used to advocate for the continuity of mental health services during and after care. While routine psychosocial screening, including suicide-specific tools, occurs at regular intervals for survivors of childhood cancer attending the Long-Term Survivor Clinics in Alberta, innovative solutions are also needed to track survivors who do not maintain long-term follow-up care within these clinics and are cared for in the community.

### 4.1. Strengths and Limitations

The strengths of this study include its population-based design, capturing all children diagnosed with cancer in Alberta over an 18-year period, and the high-quality data from the Alberta Cancer Registry. Notably, by including both overall and 5-year survivorship analyses, we captured early adverse events that are often missed when studies begin at the 5-year survival mark; this approach is critical, as survivorship starts at diagnosis [78] and treatment-related late effects may emerge earlier than previously assumed. Despite these strengths, it is important to recognize that our study is limited by the lack of detailed treatment and genetic predisposition information, as well as a relatively short period of follow-up to identify late events [79]. As well, as is common in pediatric cancer, the rarity of disease means we are limited in terms of sample size, especially in relation to conducting subgroup analyses or more complex multivariable models. Indeed, subgroup estimates of SIRs, SMRs, and AERs are included despite rare occurrence as future meta-analyses may combine multiple smaller estimates for more meaningful interpretation, though these estimates should be cautiously interpreted given their imprecision. Finally, death certificates have been shown to be imperfect, and thus some degree of misclassification is inherent in our data [80].

### 4.2. Future Directions

As this cohort will be updated every five years to add newly diagnosed survivors of childhood cancer and extend the period of follow-up, we hope to overcome some of the above limitations in the future by achieving increased statistical power. Additional follow-up and statistical power will also aid our ability to understand differences in contemporary versus historical survivors, which may become more apparent once the survivors begin to experience premature aging. In addition to SPNs and premature mortality, it will be important to understand other physical and psychosocial outcomes in this contemporary cohort of survivors of childhood cancer, as well as their healthcare utilization patterns. Finally, as two Long-Term Survivorship Clinics exist in Alberta to mitigate, diagnose, and address late effects among survivors of childhood cancer, it would be worthwhile to explore whether attendance and adherence to recommended follow-up at these clinics results in better outcomes among long-term survivors.

## 5. Conclusions

In summary, among this contemporary population-based cohort, we observed that childhood cancer survivors face a higher risk of SPNs and mortality starting at diagnosis, with continued excess risk even a decade after attaining 5-year survival. These findings add to the literature describing survivorship from both diagnosis and 5-year survival and highlight the need for the risk-based monitoring of these survivors across the life course.

## Figures and Tables

**Table 1:** Alberta Childhood Cancer Survivorship Research Program’s cohort characteristics (2001–2018).

	All Survivors n (%) or Median [IQR]			5-Year Survivors n (%) or Median [IQR]		
Characteristic	Overall (%) (n = 2581)	Dead (%) (n = 408)	SPN (%) (n = 50)	Overall (%) (n = 1385)	Dead (%) (n = 38)	SPN (%) (n = 21)
**Sex**						
Male	1354 (52.5)	234 (57.4)	25 (50.0)	740 (53.4)	23 (60.5)	7 (33.3)
Female	1227 (47.5)	174 (42.6)	25 (50.0)	645 (46.6)	15 (39.5)	14 (66.7)
**First primary neoplasm**						
Leukemias	654 (25.3)	97 (23.8)	15 (30.0)	364 (26.3)	7 (18.4)	NR
Lymphomas	385 (14.9)	31 (7.6)	14 (28.0)	233 (16.8)	7 (18.4)	8 (38.1)
CNS	624 (24.2)	127 (31.1)	5 (10.0)	314 (22.7)	11 (28.9)	NR
Neuroblastoma	161 (6.2)	27 (6.6)	NR	81 (5.8)	NR	NR
Retinoblastoma	57 (2.2)	NR	NR	38 (2.7)	NR	NR
Renal	109 (4.2)	7 (1.7)	NR	69 (5.0)	NR	NR
Hepatic	48 (1.9)	11 (2.7)	NR	19 (1.4)	NR	NR
Malignant bone	124 (4.8)	43 (10.5)	NR	56 (4.0)	NR	NR
Soft tissue	148 (5.7)	41 (10.0)	5 (10.0)	68 (4.9)	NR	NR
Germ cell	109 (4.2)	9 (2.2)	NR	61 (4.4)	NR	NR
Other epithelial	153 (5.9)	12 (2.9)	NR	78 (5.6)	NR	NR
Others/unspecified	9 (0.3)	NR	NR	NR	NR	NR
**Age at diagnosis (years)**	7.6 [2.9, 14.1]	7.0 [2.5, 14.1]	11.4 [4.7, 15.0]	8.1 [3.2, 14.4]	11.5 [4.7, 15.7]	10.3 [3.2, 14.6]
0–4	1000 (38.7)	168 (41.2)	14 (28.0)	510 (36.8)	10 (26.3)	8 (38.1)
5–9	503 (19.5)	80 (19.6)	8 (16.0)	269 (19.4)	7 (18.4)	NR
10–14	554 (21.5)	70 (17.2)	15 (30.0)	312 (22.5)	7 (18.4)	6 (28.6)
15–17	524 (20.3)	90 (22.1)	13 (26.0)	294 (21.2)	14 (36.8)	NR
**Diagnosis period**						
2001–2005	620 (24.0)	122 (29.9)	17 (34.0)	502 (36.2)	18 (47.4)	12 (57.1)
2006–2010	676 (26.2)	130 (31.9)	15 (30.0)	549 (39.6)	16 (42.1)	8 (38.1)
2011–2015	786 (30.5)	125 (30.6)	16 (32.0)	334 (24.1)	NR	NR
2016–2018	499 (19.3)	31 (7.6)	NR	NA	NA	NA
**Residence zone at diagnosis**						
South	164 (6.4)	32 (7.8)	NR	89 (6.4)	NR	NR
Calgary	915 (35.5)	144 (35.3)	22 (44.0)	471 (34.0)	10 (26.3)	11 (52.4)
Central	343 (13.3)	49 (12.0)	6 (12.0)	202 (14.6)	8 (21.1)	NR
Edmonton	815 (31.6)	121 (29.7)	15 (30.0)	440 (31.8)	12 (31.6)	5 (23.8)
North	343 (13.3)	62 (15.2)	5 (10.0)	182 (13.2)	5 (13.2)	NR
**Follow-up time (years)**	5.6 [1.9, 10.9]	1.2 [0.6, 2.4]	7.2 [3.0, 13.3]	10.3 [7.5, 13.7]	8.0 [5.9, 10.0]	13.3 [8.6, 15.6]
0–4	1196 (46.3)	370 (90.7)	20 (40.0)	NA	NA	NA
5–9	627 (24.3)	26 (6.4)	13 (26.0)	627 (45.3)	26 (68.4)	6 (28.6)
10+	758 (29.4)	12 (2.9)	17 (34.0)	758 (54.7)	12 (31.6)	15 (71.4)
**Treatment Received**						
Chemotherapy	1776 (68.8)	334 (81.9)	46 (92.0)	933 (67.4)	32 (84.2)	19 (90.5)
Surgery	1137 (44.1)	123 (30.2)	17 (34.0)	644 (46.5)	11 (29.0)	NR
Radiotherapy	807 (31.3)	224 (54.9)	32 (62.0)	422 (30.5)	29 (76.3)	14 (66.7)
Transplant	214 (8.3)	73 (17.9)	16 (32.0)	91 (6.6)	10 (26.3)	NR

SPN: subsequent primary neoplasm. NA: not applicable to 5-year survivors. NR: not reportable (i.e., observed count < 5).

**Table 2:** Observed SPNs and expected neoplasms, standardized incidence ratio (SIR), and absolute excess risk (AER) per 10,000 person-years for identified SPNs in the cohort overall and after 5-year survival.

SPN Group	All Survivors				5-Year Survivors			
	Obs/Exp	SIR (95% CI)	AER (95% CI)	AER %	Obs/Exp	SIR (95% CI)	AER (95% CI)	AER %
**All SPN types**	50/3.8	13.3 (9.8, 17.5)	27.1 (19.0, 35.3)	100.0%	21/2.1	10.0 (6.2, 15.2)	24.1 (12.6, 35.5)	100.0%
Leukemia	11/0.6	18.3 (9.2, 32.8)	6.1 (2.3, 9.9)	21.0%	0	-	-	0.0%
Non-Hodgkin Lymphoma	7/0.2	30.3 (12.2, 62.4)	3.9 (0.9, 7.0)	13.4%	NR	15.7 (1.9, 56.6)	2.4 (−1.1, 5.9)	9.5%
Hodgkin Lymphoma	NR	5.2 (0.6, 18.8)	0.9 (−0.7, 2.6)	3.1%	0	-	-	0.0%
Other Blood	NR	35.3 (9.6, 90.5)	2.3 (0.0, 4.5)	7.9%	0	-	-	0.0%
Bone and Connective Tissues	9/0.3	27.2 (12.4, 51.6)	5.1 (1.6, 8.5)	17.5%	NR	19.3 (4.0, 56.4)	3.6 (−0.7, 7.9)	14.3%
Central Nervous System	5/0.5	10.1 (3.3, 23.5)	2.6 (0.1, 5.2)	8.9%	NR	20.8 (5.7, 53.3)	4.8 (−0.1, 9.8)	19.0%
Head and Neck	NR	28.8 (3.5, 103.9)	1.1 (−0.5, 2.7)	3.8%	NR	42.5 (5.1, 153.5)	2.5 (−1.0, 6.0)	9.9%
Breast	NR	22.1 (2.7, 79.7)	1.1 (−0.5, 2.7)	3.8%	NR	23.6 (2.9, 85.3)	2.4 (−1.1, 5.9)	9.5%
Ovary	NR	12.9 (1.6, 46.7)	1.1 (−0.5, 2.7)	3.8%	NR	9.9 (0.2, 54.9)	1.1 (−1.3, 3.6)	4.4%
Endocrine Glands	NR	9.2 (2.5, 23.6)	2.1 (−0.2, 4.4)	7.2%	NR	14.2 (3.9, 36.3)	4.7 (−0.3, 9.7)	18.7%
Kidney	NR	12.0 (0.3, 66.8)	0.5 (−0.6, 1.7)	1.7%	NR	36.4 (0.9, 202.7)	1.2 (−1.3, 3.7)	4.8%
Liver	NR	25.9 (0.7, 144.4)	0.6 (−0.6, 1.7)	2.1%	0	-	-	0.0%
Ill-defined and Unknown	NR	202.0 (41.7, 590.2)	1.7 (−0.2, 3.7)	5.8%	NR	428.2 (51.9, 1546.9)	2.5 (−1.0, 6.0)	9.9%

Obs: observed; Exp: expected; SIR: standardized incidence ratio; AER: absolute excess risk; and NR: not reportable (i.e., observed count < 5).

**Table 3:** Observed SPNs and expected neoplasms, standardized incidence ratio (SIR), and absolute excess risk (AER) per 10,000 person-years in the cohort overall and after 5-year survival, by patient and cancer characteristics.

	All Children			5-Year Survivors		
Characteristic	Obs/Exp	SIR (95% CI)	AER (95% CI)	Obs/Exp	SIR (95% CI)	AER (95% CI)
**Sex**						
Male	25/2.0	12.7 (8.2, 18.8)	25.4 (14.6, 36.3)	7/1.1	6.6 (2.7, 13.6)	14.1 (1.8, 26.4)
Female	25/1.8	13.8 (9.0, 20.4)	29.1 (16.8, 41.4)	14/1.0	13.4 (7.3, 22.4)	35.8 (15.5, 56.0)
*p* for heterogeneity		0.769	0.660		0.116	0.063
**First primary neoplasm**						
Leukemias	15/0.9	17.0 (9.5, 28.1)	30.9 (14.3, 47.5)	NR	6.2 (1.3, 18.1)	11.5 (−4.0, 27.0)
Lymphomas	14/0.7	19.5 (10.6, 32.7)	48.9 (21.9, 75.8)	8/0.5	17.6 (7.6, 34.6)	58.2 (15.4, 100.9)
CNS	5/0.9	5.9 (1.9, 13.7)	10.8 (−0.6, 22.1)	NR	6.2 (1.3, 18.2)	14.5 (−5.1, 34.2)
Neuroblastoma	NR	11.0 (1.3, 39.9)	18.0 (−9.4, 45.4)	NR	14.3 (0.4, 79.7)	21.1 (−23.4, 65.5)
Retinoblastoma	NR	27.2 (3.3, 98.3)	44.6 (−19.6, 108.8)	NR	75.6 (9.2, 273.2)	99.6 (−40.3, 239.5)
Renal	NR	6.5 (0.2, 36.1)	9.8 (−12.9, 32.4)	0	-	-
Hepatic	NR	44.4 (5.4, 160.3)	78.0 (−32.6, 188.6)	0	-	-
Malignant bone	NR	11.8 (1.4, 42.6)	25.7 (−13.2, 64.6)	0	-	-
Soft tissue	5/0.2	24.9 (8.1, 58.1)	54.5 (4.7, 104.3)	NR	26.8 (5.5, 78.2)	73.0 (−12.8, 158.8)
Germ cell	NR	5.0 (0.1, 27.8)	10.8 (−15.7, 37.2)	0	-	-
Other epithelial	NR	3.6 (0.1, 20.1)	7.7 (−13.2, 28.7)	NR	6.3 (0.2, 35.1)	22.3 (−29.6, 74.1)
Other/unspecified	0	-	-	0	-	-
*p* for heterogeneity		0.113	0.139		0.100	0.107
**Age at diagnosis (years)**						
0–4	14/1.0	13.6 (7.4, 22.8)	20.5 (8.9, 32.1)	8/0.4	19.7 (8.5, 38.8)	26.3 (7.1, 45.5)
5–9	8/0.5	14.7 (6.3, 28.9)	22.1 (5.7, 38.5)	NR	6.3 (0.8, 22.6)	10.7 (−7.0, 28.4)
10–14	15/1.0	15.3 (8.6, 25.2)	36.4 (16.7, 56.1)	6/0.7	9.2 (3.4, 20.0)	28.9 (3.0, 54.8)
15–17	13/1.2	10.7 (5.7, 18.4)	33.8 (13.5, 54.1)	5/0.7	6.8 (2.2, 16.0)	27.9 (−0.7, 56.5)
*p* for trend		0.804	0.416		0.226	0.660
**Diagnosis period**						
2001–2005	17/1.9	9.1 (5.3, 14.6)	20.3 (9.5, 31.2)	12/1.4	8.6 (4.4, 15.0)	22.2 (8.0, 36.4)
2006–2010	15/1.2	13.0 (7.3, 21.5)	24.9 (11.2, 38.5)	8/0.6	12.8 (5.5, 25.3)	27.8 (6.9, 48.6)
2011–2015	16/0.7	24.4 (13.9, 39.6)	44.5 (21.8, 67.3)	NR	11.8 (0.3, 65.9)	22.7 (−25.9, 71.4)
2016–2018	NR	19.4 (2.3, 70.0)	33.6 (−15.5, 82.6)	NA	NA	NA
*p* for trend		0.050	0.214		0.674	0.905
**Follow-up time (years)**						
0–4	30/1.7	17.9 (12.1, 25.6)	30.7 (19.1, 42.3)	NA	NA	NA
5–9	9/1.2	7.7 (3.5, 14.6)	15.1 (3.8, 26.5)	9/1.2	7.6 (3.5, 14.5)	15.0 (3.7, 26.3)
10+	11/0.9	11.9 (5.9, 21.3)	38.4 (13.6, 63.2)	12/0.9	12.9 (6.7, 22.5)	42.0 (16.2, 67.7)
*p* for trend		0.053	0.115		0.232	0.036
**Residence zone at diagnosis**						
South	NR	8.0 (1.0, 28.8)	16.2 (−9.4, 41.8)	NR	7.3 (0.2, 40.4)	17.9 (−22.7, 58.4)
Calgary	22/1.2	17.6 (11.1, 26.7)	36.2 (20.2, 52.2)	11/0.7	16.1 (8.0, 28.8)	40.3 (14.9, 65.6)
Central	6/0.5	11.1 (4.1, 24.2)	22.4 (2.7, 42.0)	NR	6.6 (0.8, 23.7)	14.7 (−9.3, 38.7)
Edmonton	15/1.2	12.6 (7.0, 20.8)	25.5 (11.5, 39.5)	5/0.7	7.5 (2.4, 17.6)	17.3 (−0.2, 34.8)
North	5/0.5	9.3 (3.0, 21.8)	19.1 (0.3, 37.8)	NR	6.3 (0.8, 22.8)	15.0 (−9.7, 39.6)
*p* for heterogeneity		0.527	0.593		0.460	0.479
**Treatment Received**						
Chemotherapy	46/2.5	18.6 (13.6, 24.8)	37.3 (25.9, 48.7)	19/1.4	13.9 (8.4, 21.7)	32.7 (16.9, 48.5)
No Chemotherapy	NR	3.1 (0.84, 7.9)	5.0 (−2.3, 12.4)	NR	2.7 (0.3, 9.7)	5.1 (−6.2, 16.5)
*p* for heterogeneity		<0.001	<0.001		0.006	0.016
Surgery	17/1.8	9.5 (5.5, 15.2)	19.5 (9.1, 29.8)	NR	4.0 (1.1, 10.2)	8.3 (−2.6, 19.1)
No Surgery	33/2.0	16.7 (11.5, 23.4)	33.6 (21.4, 45.8)	17/1.1	15.4 (9.0, 24.7)	37.7 (18.5, 56.9)
*p* for heterogeneity		0.053	0.086		0.006	0.009
Radiotherapy	32/1.3	25.1 (17.2, 35.5)	58.1 (37.1, 79.1)	14/0.8	18.4 (10.1, 31.0)	54.1 (24.1, 84.1)
No radiotherapy	18/2.5	7.2 (4.3, 11.4)	13.2 (6.1, 20.3)	7/1.3	5.2 (2.1, 10.7)	10.5 (0.86, 20.1)
*p* for heterogeneity		<0.001	<0.001		0.004	0.001
Transplant	16/0.3	61.3 (35.0, 99.5)	133.9 (67.2, 200.7)	NR	28.4 (7.7, 72.7)	76.4 (−1.2, 153.9)
No transplant	34/3.5	9.7 (6.7, 13.5)	19.2 (12.0, 26.4)	17/2.0	8.6 (5.0, 13.8)	20.5 (9.5, 31.5)
*p* for heterogeneity		<0.001	<0.001		0.060	0.053

Obs: observed; Exp: expected; SIR: standardized incidence ratio; AER: absolute excess risk; SPN: subsequent primary neoplasm; CNS: central nervous system; *p*: *p*-value; and NR: not reportable (i.e., observed count < 5). NA: not applicable to 5-year survivors.

**Table 4:** Observed and expected deaths, standardized mortality ratio (SMR), and absolute excess risk (AER) per 10,000 person-years for identified causes of death in the cohort overall and 5-year survivors.

Cause of Death	All Survivors				5-Year Survivors			
	Observed/Expected	SMR (95% CI)	AER (95% CI)	AER %	Observed/Expected	SMR (95% CI)	AER (95% CI)	AER %
**All causes**	408/6.5	62.5 (56.5, 68.8)	233.9 (210.8, 257.0)	100.0%	38/3.5	10.9 (7.7, 15.0)	43.8 (28.4, 59.1)	100.0%
**Neoplastic**	380/0.5	757.9 (683.6, 838.1)	221.1 (198.8, 243.4)	94.5%	29/0.3	111.9 (74.9, 160.7)	36.4 (23.0, 49.8)	83.1%
Recurrence or progression	361/0.0	-	210.3 (188.6, 232.0)	89.9%	23/0.0	-	29.1 (17.2, 41.1)	66.4%
Subsequent primary neoplasm	19/0.5	37.9 (22.8, 59.2)	10.8 (5.8, 15.8)	4.6%	6/0.3	23.2 (8.5, 50.4)	7.3 (1.2, 13.4)	16.7%
**Non-neoplastic**	28/6.0	4.6 (3.1, 6.7)	12.8 (6.8, 18.8)	5.5%	9/3.2	2.8 (1.3, 5.3)	7.3 (−0.1, 14.8)	16.7%
Health-related ^1^	17/1.8	9.3 (5.4, 14.8)	8.8 (4.1, 13.5)	3.8%	NR	1.6 (0.0, 9.1)	0.5 (−2.0, 3.0)	1.1%
External ^2^	11/4.2	2.6 (1.3, 4.7)	4.0 (0.2, 7.8)	1.7%	8/2.6	3.1 (1.3, 6.1)	6.8 (−0.2, 13.9)	15.5%
Suicide	5/1.2	4.0 (1.3, 9.4)	2.2 (−0.4, 4.7)	0.9%	NR	5.0 (1.4, 12.7)	4.0 (−0.9, 9.0)	9.1%

SMR: standardized mortality ratio; AER, absolute excess risk; and NR, not reportable (i.e., observed count < 5). ^1^ Health-related deaths include circulatory, respiratory, nervous, infection, digestive, perinatal, endocrine, genitourinary, musculoskeletal, mental, blood, and other deaths. ^2^ External deaths include accidents such as falls, drownings or fire, and suicide.

**Table 5:** Observed and expected cause-specific deaths, standardized mortality ratio (SMR), and absolute excess risk (AER) per 10,000 person-years in the cohort overall, by patient and cancer characteristics.

	Recurrence/Progression			SPN				Non-Neoplastic Causes			
Characteristic	Obs/Exp	AER (95% CI)	AER %	Obs/Exp	SMR (95% CI)	AER (95% CI)	AER %	Obs/Exp	SMR (95% CI)	AER (95% CI)	AER %
**Sex**											
Male	206/0.0	225.6 (194.8, 256.5)	89.7%	10/0.3	33.8 (16.2, 62.1)	10.6 (3.8, 17.4)	4.2%	18/4.1	4.4 (2.6, 7.0)	15.3 (6.1, 24.4)	6.1%
Female	155/0.0	192.9 (162.5, 223.3)	90.2%	9/0.2	43.9 (20.1, 83.2)	10.9 (3.6, 18.3)	5.1%	10/2.0	5.1 (2.5, 9.4)	10.0 (2.3, 17.7)	4.7%
*p* for heterogeneity		0.139			0.571	0.950			0.713	0.390	
**First primary neoplasm**											
Leukemias	84/0.0	183.0 (143.8, 222.1)	87.9%	8/0.1	63.9 (27.6, 125.9)	17.2 (5.1, 29.2)	8.3%	5/1.3	4.0 (1.3, 9.3)	8.2 (−1.4, 17.7)	3.9%
Lymphomas	19/0.0	68.6 (37.8, 99.5)	64.0%	NR	43.5 (11.9, 111.5)	14.1 (0.0, 28.3)	13.2%	8/1.2	6.5 (2.8, 12.8)	24.4 (4.4, 44.5)	22.8%
CNS	120/0.0	309.2 (253.9, 364.5)	95.6%	NR	17.5 (2.1, 63.1)	4.9 (−2.3, 12.0)	1.5%	5/1.3	3.8 (1.2, 8.8)	9.5 (−1.8, 20.8)	2.9%
Neuroblastoma	24/0.0	236.7 (142.0, 331.3)	90.2%	NR	39.4 (1.0, 219.8)	9.6 (−9.7, 28.9)	3.7%	NR	5.3 (0.6, 19.1)	16.0 (−11.3, 43.3)	6.1%
Retinoblastoma	NR	23.0 (−22.1, 68.1)	53.9%	NR	99.9 (2.5, 556.8)	22.8 (−22.3, 67.9)	53.4%	0	-	-	0.0%
Renal	7/0.0	80.2 (20.8, 139.6)	100.0%	0	-	-	0.0%	0	-	-	0.0%
Hepatic	10/0.0	381.6 (145.1, 618.2)	91.7%	0	-	-	0.0%	NR	11.7 (0.3, 65.3)	34.9 (−39.9, 109.7)	8.4%
Malignant bone	41/0.0	572.1 (397.0, 747.3)	96.0%	NR	45.9 (1.2, 255.9)	13.7 (−13.7, 41.0)	2.3%	NR	3.6 (0.1, 20.1)	10.1 (−17.3, 37.4)	1.7%
Soft tissue	36/0.0	404.7 (272.5, 536.9)	88.7%	NR	72.9 (8.8, 263.3)	22.2 (−9.0, 53.3)	4.9%	NR	8.1 (1.7, 23.6)	29.5 (−8.6, 67.7)	6.5%
Germ cell	8/0.0	107.6 (33.0, 182.1)	93.0%	0	-	-	0.0%	NR	2.7 (0.1, 15.0)	8.5 (−17.9, 34.8)	7.3%
Other epithelial	10/0.0	106.7 (40.6, 172.9)	86.4%	0	-	-	0.0%	NR	5.0 (0.6, 18.0)	17.1 (−12.5, 46.6)	13.8%
Other/unspecified	NR	184.7 (−177.3, 546.6)	100.0%	0	-	-	0.0%	0	-	-	0.0%
*p* for heterogeneity		<0.001			0.411	0.468			0.866	0.690	
**Age at diagnosis (years)**											
0–4	144/0.0	227.1 (190.0, 264.2)	86.6%	9/0.2	59.2 (27.1, 112.3)	14.0 (4.7, 23.2)	5.3%	15/1.5	9.9 (5.5, 16.3)	21.3 (9.3, 33.2)	8.1%
5–9	76/0.0	223.0 (172.9, 273.1)	96.0%	NR	11.4 (0.3, 63.4)	2.7 (−3.1, 8.4)	1.2%	NR	4.1 (0.9, 12.0)	6.7 (−3.3, 16.6)	2.9%
10–14	64/0.0	163.8 (123.7, 204.0)	94.0%	NR	23.8 (4.9, 69.7)	7.4 (−1.3, 16.0)	4.2%	NR	1.7 (0.3, 4.9)	3.1 (−5.6, 11.8)	1.8%
15–17	77/0.0	219.5 (170.4, 268.5)	87.7%	6/0.1	44.2 (16.2, 96.3)	16.7 (3.0, 30.4)	6.7%	7/2.0	3.5 (1.4, 7.2)	14.2 (−0.6, 29.0)	5.7%
*p* for trend		0.309			0.524	0.877			0.004	0.129	
**Diagnosis period**											
2001–2005	109/0.0	144.8 (117.7, 172.0)	91.8%	6/0.2	24.4 (8.9, 53.1)	7.6 (1.3, 14.0)	4.8%	7/3.0	2.3 (0.9, 4.8)	5.3 (−1.6, 12.2)	3.4%
2006–2010	114/0.0	203.6 (166.3, 241.0)	89.0%	7/0.2	45.5 (18.3, 93.8)	12.2 (3.0, 21.5)	5.3%	9/1.7	5.2 (2.4, 9.8)	13.0 (2.5, 23.5)	5.7%
2011–2015	110/0.0	316.6 (257.5, 375.8)	88.8%	5/0.1	56.4 (18.3, 131.6)	14.1 (1.5, 26.8)	4.0%	10/1.1	9.5 (4.5, 17.4)	25.7 (7.9, 43.6)	7.2%
2016–2018	28/0.0	493.7 (310.9, 676.6)	91.0%	NR	78.6 (2.0, 437.9)	17.4 (−17.2, 52.0)	3.2%	NR	9.3 (1.1, 33.6)	31.5 (−17.4, 80.4)	5.8%
*p* for trend		<0.001			0.114	0.267			0.004	0.011	
**Follow-up time (years)**											
0–4	338/0.0	364.5 (325.6, 403.3)	92.1%	13/0.2	53.7 (28.6, 91.8)	13.8 (6.1, 21.4)	3.5%	19/2.8	6.7 (4.1, 10.5)	17.4 (8.2, 26.7)	4.4%
5–9	19/0.0	36.4 (20.0, 52.8)	79.0%	NR	19.3 (4.0, 56.4)	5.5 (−1.1, 12.0)	11.9%	NR	2.3 (0.6, 5.8)	4.3 (−3.2, 11.8)	9.3%
10+	NR	15.0 (0.3, 29.6)	38.4%	NR	29.0 (6.0, 84.6)	10.8 (−1.9, 23.5)	27.6%	5/1.4	3.5 (1.1, 8.1)	13.3 (−3.1, 29.7)	34.0%
*p* for trend		<0.001			0.149	0.378			0.066	0.220	
**Residence zone at diagnosis**											
South	28/0.0	258.1 (162.5, 353.8)	88.6%	NR	30.9 (0.8, 172.1)	8.9 (−9.1, 27.0)	3.1%	NR	7.9 (1.6, 23.1)	24.2 (−7.1, 55.5)	8.3%
Calgary	128/0.0	220.7 (182.4, 258.9)	90.2%	9/0.2	54.1 (24.7, 102.7)	15.2 (5.1, 25.4)	6.2%	7/1.9	3.6 (1.5, 7.5)	8.7 (−0.2, 17.7)	3.6%
Central	43/0.0	175.0 (122.7, 227.2)	89.5%	NR	27.9 (3.4, 100.7)	7.8 (−3.4, 19.1)	4.0%	NR	4.6 (1.3, 11.8)	12.8 (−3.2, 28.7)	6.5%
Edmonton	109/0.0	199.9 (162.4, 237.4)	91.7%	NR	25.0 (6.8, 64.1)	7.0 (−0.1, 14.2)	3.2%	8/2.0	4.1 (1.8, 8.0)	11.1 (0.9, 21.2)	5.1%
North	53/0.0	225.4 (164.7, 286.1)	86.8%	NR	42.5 (8.8, 124.1)	12.5 (−2.0, 26.9)	4.8%	6/0.9	6.7 (2.5, 14.7)	21.7 (1.3, 42.2)	8.4%
*p* for heterogeneity		0.482			0.718	0.735			0.723	0.657	
**Treatment Received**											
Chemotherapy	297/0.0	251.9 (223.3, 280.6)	90.00%	19/0.3	56.0 (33.4, 87.5)	15.8 (8.6, 23.1)	5.7%	18/4.0	4.5 (2.7, 7.1)	11.9 (4.8, 18.9)	4.3%
No Chemotherapy	64/0.0	119.1 (89.9, 148.3)	89.10%	0	-	-	-	10/2.0	4.9 (2.4, 9.0)	14.8 (3.3, 26.4)	11.10%
*p* for heterogeneity		<0.001			<0.001	<0.001			0.831	0.664	
Surgery	106/0.0	134.7 (109.1, 160.4)	91.2%	6/0.2	25.9 (9.5, 56.4)	7.3 (1.2, 13.4)	4.8%	11/3.0	3.7 (1.9, 6.7)	10.3 (2.0, 18.5)	4.0%
No Surgery	255/0.0	274.2 (240.6, 308.0)	90.50%	13/0.3	48.2 (25.6, 82.4)	13.7 (6.1, 21.3)	4.5%	17/3.1	5.5 (3.2, 8.8)	15.0 (6.3, 23.6)	5.0%
*p* for heterogeneity		<0.001			0.195	0.206			0.318	0.445	
Radiotherapy	199/0.0	370.8 (319.3, 422.3)	89.60%	14/0.2	84.2 (46.0, 141.2)	25.8 (12.1, 39.4)	6.20%	11/2.0	5.5 (2.8, 9.8)	16.8 (4.7, 28.8)	4.10%
No radiotherapy	162/0.0	137.3 (116.2, 158.5)	90.10%	5/0.3	14.9 (4.8, 34.8)	4.0 (0.2, 7.7)	2.60%	17/4.0	4.2 (2.5, 6.8)	11.0 (4.1, 17.8)	7.2%
*p* for heterogeneity		<0.001			<0.001	<0.001			0.497	0.396	
Transplant	63/0.0	511.6 (385.3, 637.9)	86.90%	7/0.0	186.5 (75.0, 384.2)	56.5 (14.4, 98.6)	9.60%	NR	6.5 (1.3, 19.1)	20.6 (−6.9, 48.2)	3.50%
No transplant	298/0.0	187.0 (165.8, 208.3)	90.8%	12/0.5	25.9 (13.4, 45.2)	7.2 (3.0, 11.5)	3.50%	25/5.6	4.5 (2.9, 6.6)	12.2 (6.0, 18.3)	5.90%
*p* for heterogeneity		<0.001			<0.001	<0.001			0.560	0.510	

Obs: observed; Exp: expected; SMR: standardized mortality ratio; AER: absolute excess risk; SPN: subsequent primary neoplasm; CNS: central nervous system; *p*: *p*-value; and NR: not reportable (i.e., observed count < 5).

## Data Availability

The data underlying this article cannot be shared publicly as they contain semi-identifiable patient information. Additional information on summary counts can be requested by contacting the corresponding author.

## Supplementary Materials

The following supporting information can be downloaded at: https://www.mdpi.com/article/10.3390/cancers18040694/s1, Table S1: Observed and expected all cause and neoplastic deaths, with corresponding standard-ized mortality ratios (SMRs) and absolute excess risks (AERs) per 10,000 person-years, in the cohort overall, by patient and cancer characteristics; Table S2: Observed and expected all cause and cause-specific deaths, with corresponding standardized mortality ratios (SMRs) and absolute excess risks (AERs) per 10,000 person-years, among 5-year survivors, by patient and cancer char-acteristics.

## Abbreviations

AER	Absolute excess risk
CI	Confidence interval
CNS	Central nervous system
ICCC-3	International Classification of Childhood Cancer, third revision
ICD	International Classification of Diseases
ICD-O	International Classification of Diseases for Oncology
NCI	National Cancer Institute
SIR	Standardized incidence ratio
SMR	Standardized mortality ratio
SPN	Subsequent primary neoplasm
