# Supplementary material for: Subsequent Primary Neoplasms and Mortality Among Survivors of Childhood Cancer in Alberta, Canada
Source: Cancers (Basel). 2026 Feb 20;18(4):694. doi: 10.3390/cancers18040694 (PMC12939232; doi:10.3390/cancers18040694)
Supplement: Supplementary file 1 [file cancers-18-00694-s001.zip › cancers-4136087-supplementary.pdf]

**Table S1. Observed and expected all cause and neoplastic deaths, with corresponding standardized mortality ratios (SMRs) and absolute excess risks (AERs) per 10,000 person-years, in the cohort overall, by patient and cancer characteristics.**

| | All causes | | | Neoplastic causes | | | |
|---|---|---|---|---|---|---|---|
| **Characteristic** | **Obs/ Exp** | **SMR (95% CI)** | **AER (95% CI)** | **Obs/ Exp** | **SMR (95% CI)** | **AER (95% CI)** | **AER %** |
| **Sex** | | | | | | | |
| Male | 234/4.4 | 53.5 (46.9, 60.9) | 251.5 (218.7, 284.4) | 216/0.3 | 729.4 (635.3, 833.4) | 236.3 (204.7, 267.8) | 94.0% |
| Female | 174/2.2 | 80.5 (69.0, 93.4) | 213.9 (181.7, 246.0) | 164/0.2 | 799.1 (681.4, 931.2) | 203.8 (172.6, 235.1) | 95.3% |
| *p* for heterogeneity | | <0.001 | 0.109 | | 0.379 | 0.153 | |
| **First primary neoplasm** | | | | | | | |
| Leukemias | 97/1.4 | 70.4 (57.1, 85.8) | 208.3 (166.2, 250.3) | 92/0.1 | 734.8 (592.4, 901.2) | 200.1 (159.2, 241.0) | 96.1% |
| Lymphomas | 31/1.3 | 23.4 (15.9, 33.2) | 107.2 (67.8, 146.6) | 23/0.1 | 250.3 (158.7, 375.6) | 82.8 (48.8, 116.7) | 77.2% |
| CNS | 127/1.4 | 88.2 (73.5, 105.0) | 323.5 (266.6, 380.4) | 122/0.1 | 1064.9 (884.4, 1271.5) | 314.0 (258.3, 369.8) | 97.1% |
| Neuroblastoma[4] | 27/0.4 | 66.8 (44.0, 97.2) | 262.3 (161.8, 362.7) | 25/0.0 | 986.0 (638.1, 1455.6) | 246.3 (149.6, 342.9) | 93.9% |
| Retinoblastoma | NR | 13.8 (1.7, 50.0) | 42.7 (-21.1, 106.5) | NR | 199.9 (24.2, 722.0) | 45.8 (-18.0, 109.6) | 100.0% |
| Renal | 7/0.2 | 35.3 (14.2, 72.8) | 77.9 (18.5, 137.3) | 7/0.0 | 315.2 (126.7, 649.4) | 79.9 (20.5, 139.3) | 100.0% |
| Hepatic | 11/0.1 | 119.3 (59.6, 213.5) | 416.3 (168.2, 664.4) | 10/0.0 | 1469.0 (704.4, 2701.5) | 381.4 (144.8, 617.9) | 91.6% |
| Malignant bone | 43/0.3 | 143.9 (104.1, 193.8) | 595.9 (416.5, 775.2) | 42/0.0 | 1928.8 (1390.1, 2607.1) | 585.8 (408.5, 763.1) | 98.3% |
| Soft tissue | 41/0.4 | 102.8 (73.8, 139.5) | 456.4 (315.3, 597.5) | 38/0.0 | 1385.1 (980.2, 1901.2) | 426.9 (291.0, 562.7) | 93.5% |
| Germ cell | 9/0.4 | 22.7 (10.4, 43.2) | 115.7 (36.6, 194.7) | 8/0.0 | 325.0 (140.3, 640.5) | 107.2 (32.7, 181.8) | 92.7% |
| Other epithelial | 12/0.4 | 27.8 (14.3, 48.5) | 123.5 (51.0, 195.9) | 10/0.0 | 332.2 (159.3, 611.0) | 106.4 (40.3, 172.6) | 86.2% |
| Other/unspecified | NR | 44.4 (1.1, 247.6) | 180.5 (-181.4, 542.5) | NR | 696.5 (17.6, 3880.8) | 184.4 (-177.5, 546.4) | 100.0% |
| *p* for heterogeneity | | <0.001 | <0.001 | | <0.001 | <0.001 | |
| **Age at diagnosis (years)** | | | | | | | |
| 0 – 4 | 168/1.7 | 100.9 (86.2, 117.3) | 262.3 (222.2, 302.3) | 153/0.2 | 1005.8 (852.8, 1178.4) | 241.0 (202.8, 279.3) | 91.9% |
| 5 – 9 | 80/0.8 | 98.1 (77.8, 122.1) | 232.4 (180.9, 283.8) | 77/0.1 | 876.7 (691.9, 1095.7) | 225.7 (175.2, 276.1) | 97.1% |
| 10 – 14 | 70/1.9 | 36.8 (28.7, 46.4) | 174.3 (132.3, 216.3) | 67/0.1 | 532.5 (412.7, 676.3) | 171.2 (130.1, 212.2) | 98.2% |
| 15 – 17 | 90/2.1 | 41.9 (33.7, 51.5) | 250.4 (197.4, 303.4) | 83/0.1 | 612.0 (487.4, 758.6) | 236.2 (185.3, 287.1) | 94.3% |
| *p* for trend | | <0.001 | 0.200 | | <0.001 | 0.339 | |
| **Diagnosis period** | | | | | | | |
| 2001-2005 | 122/3.3 | 37.4 (31.0, 44.6) | 157.8 (129.0, 186.6) | 115/0.2 | 467.2 (385.7, 560.8) | 152.5 (124.6, 180.4) | 96.6% |
| 2006-2010 | 130/1.9 | 68.6 (57.3, 81.4) | 228.8 (188.9, 268.7) | 121/0.2 | 786.5 (652.6, 939.8) | 215.9 (177.4, 254.4) | 94.4% |
| 2011-2015 | 125/1.1 | 109.3 (91.0, 130.2) | 356.5 (293.4, 419.6) | 115/0.1 | 1297.1 (1070.9, 1556.9) | 330.8 (270.3, 391.3) | 92.8% |
| 2016-2018 | 31/0.2 | 136.3 (92.6, 193.4) | 542.6 (350.2, 735.1) | 29/0.0 | 2279.1 (1526.4, 3273.2) | 511.2 (325.0, 697.3) | 94.2% |
| *p* for trend | | <0.001 | <0.001 | | <0.001 | <0.001 | |
| **Follow-up time (years)** | | | | | | | |
| 0 – 4 | 370/3.1 | 121.0 (108.9, 133.9) | 395.7 (355.0, 436.3) | 351/0.2 | 1448.8 (1301.2, 1608.6) | 378.2 (338.6, 417.8) | 95.6% |
| 5 – 9 | 26/1.9 | 13.5 (8.8, 19.7) | 46.1 (27.0, 65.3) | 22/0.2 | 141.5 (88.7, 214.2) | 41.9 (24.2, 59.5) | 90.9% |
| 10+ | 12/1.5 | 7.8 (4.0, 13.6) | 39.1 (13.7, 64.5) | 7/0.1 | 67.6 (27.2, 139.2) | 25.8 (6.4, 45.2) | 66.0% |
| *p* for trend | | <0.001 | <0.001 | | <0.001 | <0.001 | |
| **Residence zone at diagnosis** | | | | | | | |
| South | 32/0.4 | 77.8 (53.2, 109.8) | 291.2 (189.0, 393.4) | 29/0.0 | 896.0 (600.0, 1286.7) | 267.1 (169.8, 364.4) | 91.7% |
| Calgary | 144/2.1 | 68.6 (57.9, 80.8) | 244.6 (204.1, 285.2) | 137/0.2 | 823.2 (691.1, 973.2) | 235.9 (196.3, 275.4) | 96.4% |
| Central | 49/0.9 | 52.3 (38.7, 69.1) | 195.6 (139.7, 251.4) | 45/0.1 | 627.2 (457.5, 839.3) | 182.8 (129.3, 236.3) | 93.5% |
| Edmonton | 121/2.1 | 57.1 (47.4, 68.2) | 218.0 (178.5, 257.6) | 113/0.2 | 707.2 (582.8, 850.3) | 206.9 (168.7, 245.2) | 94.9% |
| North | 62/1.0 | 64.5 (49.5, 82.7) | 259.6 (194.0, 325.3) | 56/0.1 | 792.8 (598.9, 1029.5) | 237.9 (175.5, 300.3) | 91.6% |
| *p* for heterogeneity | | 0.248 | 0.343 | | 0.398 | 0.398 | |
| **Treatment Received** | | | | | | | |
| Chemotherapy | 296/4.1 | 71.5 (63.6, 80.1) | 258.3 (228.5, 288.2) | 279/0.3 | 859.2 (761.3, 966.1) | 246.7 (217.7, 275.6) | 95.5% |
| No Chemotherapy | 112/2.4 | 46.8 (38.5, 56.3) | 186.8 (151.5, 222.2) | 101/0.2 | 571.7 (465.7, 694.7) | 171.9 (138.3, 205.4) | 92.0% |
| *p* for heterogeneity | | <0.001 | 0.003 | | <0.001 | 0.001 | |
| Surgery | 159/3.2 | 48.9 (41.6, 57.2) | 184.9 (155.6, 214.3) | 151/0.2 | 615.4 (521.1, 721.7) | 179.0 (150.4, 207.6) | 96.8% |
| No Surgery | 249/3.3 | 75.8 (66.7, 85.9) | 281.0 (245.7, 316.4) | 229/0.3 | 894.5 (782.4, 1018.2) | 261.6 (227.7, 295.5) | 93.1% |
| *p* for heterogeneity | | <0.001 | <0.001 | | <0.001 | <0.001 | |
| Radiotherapy | 168/1.5 | 114.5 (97.8, 133.2) | 442.4 (374.9, 509.9) | 162/0.1 | 1402.7 (1195.0, 1636.1) | 430.1 (363.8, 496.3) | 97.2% |
| No radiotherapy | 240/5.1 | 47.4 (41.6, 53.8) | 175.3 (152.7, 198.0) | 218/0.4 | 564.9 (492.4, 645.1) | 162.4 (140.8, 184.0) | 92.6% |
| *p* for heterogeneity | | <0.001 | <0.001 | | <0.001 | <0.001 | |
| Transplant | 56/0.3 | 178.4 (134.8, 231.7) | 609.8 (449.2, 770.4) | 55/0.0 | 2087.8 (1572.8, 2717.6) | 602.0 (442.8, 761.2) | 98.7% |
| No transplant | 352/6.2 | 56.6 (50.9, 62.8) | 212.8 (190.1, 235.4) | 325/0.5 | 684.1 (611.8, 762.7) | 199.7 (177.9, 221.4) | 93.8% |
| *p* for heterogeneity | | <0.001 | <0.001 | | <0.001 | <0.001 | |

Obs, observed; Exp, expected; SMR, standardized mortality ratio; AER, absolute excess risk; SPN, subsequent primary neoplasm; CNS, central nervous system; *p*, *p*-value; NR, not reportable (i.e. observed count <5).

**Table S2. Observed and expected all cause and cause-specific deaths, with corresponding standardized mortality ratios (SMRs) and absolute excess risks (AERs) per 10,000 person-years, among 5-year survivors, by patient and cancer characteristics.**

| | All causes | | | Neoplastic causes | | | | Recurrence/progression | | | SPN | | | | Non-neoplastic causes | | | |
|---|---|---|---|---|---|---|---|---|---|---|---|---|---|---|---|---|---|---|
| **Characteristic** | **Obs/Exp** | **SMR (95% CI)** | **AER (95% CI)** | **Obs/Exp** | **SMR (95% CI)** | **AER (95% CI)** | **AER %** | **Obs/Exp** | **AER (95% CI)** | **AER %** | **Obs/Exp** | **SMR (95% CI)** | **AER (95% CI)** | **AER %** | **Obs/Exp** | **SMR (95% CI)** | **AER (95% CI)** | **AER %** |
| **Sex** | | | | | | | | | | | | | | | | | | |
| Male | 23/2.4 | 9.4 (6.0, 14.1) | 48.5 (26.3, 70.6) | 16/0.1 | 107.3 (61.3, 174.3) | 37.4 (18.9, 55.9) | 77.1% | 13/0.0 | 30.7 (14.0, 47.3) | 63.3% | NR | 20.1 (4.1, 58.8) | 6.7 (-1.3, 14.7) | 13.8% | 7/2.3 | 3.1 (1.2, 6.3) | 11.1 (-1.1, 23.3) | 22.9% |
| Female | 15/1.0 | 14.6 (8.2, 24.0) | 38.3 (17.5, 59.1) | 13/0.1 | 118.1 (62.9, 202.0) | 35.3 (16.0, 54.7) | 92.2% | 10/0.0 | 27.4 (10.4, 44.4) | 71.5% | NR | 27.3 (5.6, 79.7) | 7.9 (-1.4, 17.2) | 20.6% | NR | 2.2 (0.3, 7.9) | 3.0 (-4.6, 10.6) | 7.8% |
| *p* for heterogeneity | | 0.196 | 0.512 | | 0.797 | 0.880 | | | 0.789 | | | 0.710 | 0.848 | | | 0.664 | 0.263 | |
| **First primary neoplasm** | | | | | | | | | | | | | | | | | | |
| Leukemias | 7/0.8 | 8.7 (3.5, 18.0) | 28.2 (4.6, 51.9) | 5/0.1 | 77.4 (25.1, 180.7) | 22.5 (2.5, 42.4) | 79.8% | 5/0.0 | 22.8 (2.8, 42.7) | 80.9% | 0 | - | - | 0.0% | NR | 2.7 (0.3, 9.8) | 5.8 (-6.9, 18.4) | 20.6% |
| Lymphomas | 7/0.8 | 8.5 (3.4, 17.5) | 47.0 (7.5, 86.4) | NR | 57.9 (11.9, 169.3) | 22.4 (-3.4, 48.2) | 47.7% | NR | 7.6 (-7.3, 22.5) | 16.2% | NR | 38.6 (4.7, 139.5) | 14.8 (-6.3, 35.9) | 31.5% | NR | 5.2 (1.4, 13.3) | 24.6 (-5.2, 54.4) | 52.3% |
| CNS | 11/0.8 | 13.6 (6.8, 24.4) | 58.5 (21.2, 95.7) | 10/0.1 | 170.4 (81.7, 313.4) | 57.0 (21.5, 92.6) | 97.4% | 8/0.0 | 45.9 (14.1, 77.7) | 78.5% | NR | 34.1 (4.1, 123.1) | 11.1 (-4.8, 27.0) | 19.0% | NR | 1.3 (0.0, 7.4) | 1.4 (-9.8, 12.7) | 2.4% |
| Neuroblastoma[4] | NR | 34.3 (7.1, 100.3) | 66.0 (-10.9, 143.0) | NR | 274.1 (56.5, 801.1) | 67.8 (-9.2, 144.8) | 100.0% | NR | 68.0 (-9.0, 145.0) | 100.0% | 0 | - | - | 0.0% | 0 | - | - | 0.0% |
| Retinoblastoma | NR | 33.7 (0.9, 187.5) | 48.3 (-49.3, 146.0) | NR | 219.1 (5.5, 1220.5) | 49.6 (-48.1, 147.2) | 100.0% | 0 | - | 0.0% | NR | 219.1 (5.5, 1220.5) | 49.6 (-48.1, 147.2) | 100.0% | 0 | - | - | 0.0% |
| Renal | 0 | - | - | 0 | - | - | 0.0% | 0 | - | 0.0% | 0 | - | - | 0.0% | 0 | - | - | 0.0% |
| Hepatic | NR | 38.4 (1.0, 214.2) | 82.8 (-83.8, 249.5) | NR | 347.7 (8.8, 1937.3) | 84.8 (-81.9, 251.4) | 100.0% | NR | 85.0 (-81.6, 251.7) | 100.0% | 0 | - | - | 0.0% | 0 | - | - | 0.0% |
| Malignant bone | NR | 19.0 (3.9, 55.6) | 97.1 (-18.9, 213.0) | NR | 294.9 (60.8, 862.0) | 102.1 (-13.8, 218.0) | 100.0% | NR | 102.5 (-13.5, 218.4) | 100.0% | 0 | - | - | 0.0% | 0 | - | - | 0.0% |
| Soft tissue | NR | 14.3 (2.9, 41.7) | 69.5 (-15.1, 154.1) | NR | 143.7 (17.4, 519.1) | 49.5 (-19.6, 118.6) | 71.2% | NR | 24.9 (-23.9, 73.8) | 35.8% | NR | 71.8 (1.8, 400.3) | 24.6 (-24.3, 73.4) | 35.4% | NR | 5.1 (0.1, 28.4) | 20.0 (-28.8, 68.9) | 28.8% |

| | All causes | | | Neoplastic causes | | | | Recurrence/progression | | | SPN | | | | Non-neoplastic causes | | | |
|---|---|---|---|---|---|---|---|---|---|---|---|---|---|---|---|---|---|---|
| **Characteristic** | **Obs/Exp** | **SMR (95% CI)** | **AER (95% CI)** | **Obs/Exp** | **SMR (95% CI)** | **AER (95% CI)** | **AER %** | **Obs/Exp** | **AER (95% CI)** | **AER %** | **Obs/Exp** | **SMR (95% CI)** | **AER (95% CI)** | **AER %** | **Obs/Exp** | **SMR (95% CI)** | **AER (95% CI)** | **AER %** |
| Germ cell | 0 | - | - | 0 | - | - | 0.0% | 0 | - | 0.0% | 0 | - | - | 0.0% | 0 | - | - | 0.0% |
| Other epithelial | NR | 9.1 (1.1, 33.0) | 47.0 (-26.1, 120.1) | NR | 63.8 (1.6, 355.2) | 26.0 (-25.7, 77.7) | 55.3% | NR | 26.4 (-25.3, 78.1) | 56.2% | 0 | - | - | 0.0% | NR | 4.9 (0.1, 27.4) | 21.0 (-30.7, 72.7) | 44.7% |
| Other/ unspecified | 0 | - | - | 0 | - | - | 0.0% | 0 | - | 0.0% | 0 | - | - | 0.0% | 0 | - | - | 0.0% |
| *p* for heterogeneity | | 0.235 | 0.400 | | 0.163 | 0.239 | | | 0.128 | | | 0.499 | 0.417 | | | 0.895 | 0.848 | |
| **Age at diagnosis (years)** | | | | | | | | | | | | | | | | | | |
| 0 – 4 | 10/0.5 | 20.0 (9.6, 36.9) | 32.8 (11.4, 54.1) | 9/0.1 | 133.8 (61.2, 253.9) | 30.8 (10.5, 51.1) | 93.9% | 5/0.0 | 17.2 (2.1, 32.4) | 52.4% | NR | 59.4 (16.2, 152.2) | 13.6 (0.0, 27.1) | 41.5% | NR | 2.3 (0.1, 12.9) | 2.0 (-4.8, 8.7) | 6.1% |
| 5 – 9 | 7/0.6 | 11.7 (4.7, 24.0) | 40.4 (7.7, 73.2) | 5/0.0 | 110.1 (35.8, 257.0) | 31.3 (3.6, 59.0) | 77.5% | 5/0.0 | 31.6 (3.9, 59.3) | 78.2% | 0 | - | - | 0.0% | NR | 3.6 (0.4, 13.0) | 9.1 (-8.4, 26.6) | 22.5% |
| 10 – 14 | 7/1.3 | 5.5 (2.2, 11.4) | 30.6 (2.9, 58.4) | 5/0.1 | 67.5 (21.9, 157.5) | 26.3 (2.9, 49.8) | 85.9% | 5/0.0 | 26.7 (3.3, 50.2) | 87.3% | 0 | - | - | 0.0% | NR | 1.7 (0.2, 6.1) | 4.3 (-10.5, 19.1) | 14.1% |
| 15 – 17 | 14/1.1 | 12.6 (6.9, 21.2) | 83.9 (36.2, 131.6) | 10/0.1 | 138.2 (66.3, 254.1) | 64.6 (24.3, 104.9) | 77.0% | 8/0.0 | 52.0 (16.0, 88.1) | 62.0% | NR | 27.6 (3.3, 99.8) | 12.5 (-5.5, 30.6) | 14.9% | NR | 3.9 (1.1, 9.9) | 19.3 (-6.2, 44.8) | 23.0% |
| *p* for trend | | 0.234 | 0.069 | | 0.877 | 0.177 | | | 0.070 | | | 0.212 | 0.534 | | | 0.719 | 0.164 | |
| **Diagnosis period** | | | | | | | | | | | | | | | | | | |
| 2001-2005 | 18/2.3 | 7.8 (4.6, 12.3) | 32.5 (15.3, 49.8) | 14/0.2 | 82.4 (45.0, 138.2) | 28.7 (13.5, 43.9) | 88.3% | 11/0.0 | 22.8 (9.3, 36.3) | 70.2% | NR | 17.7 (3.6, 51.6) | 5.9 (-1.2, 12.9) | 18.2% | NR | 1.9 (0.5, 4.8) | 3.8 (-4.3, 12.0) | 11.7% |
| 2006-2010 | 16/1.0 | 16.0 (9.1, 25.9) | 56.2 (26.8, 85.6) | 13/0.1 | 162.2 (86.4, 277.4) | 48.5 (21.9, 75.0) | 86.3% | 10/0.0 | 37.5 (14.3, 60.7) | 66.7% | NR | 37.4 (7.7, 109.4) | 10.9 (-1.8, 23.7) | 19.4% | NR | 3.3 (0.7, 9.5) | 7.8 (-4.9, 20.5) | 13.9% |
| 2011-2015 | NR | 25.6 (7.0, 65.5) | 95.2 (-1.9, 192.3) | NR | 221.2 (26.8, 799.1) | 49.3 (-19.3, 118.0) | 51.8% | NR | 49.5 (-19.1, 118.2) | 52.0% | 0 | - | - | 0.0% | NR | 13.6 (1.6, 49.0) | 45.9 (-22.8, 114.6) | 48.2% |
| *p* for trend | | 0.011 | 0.049 | | 0.056 | 0.197 | | | 0.189 | | | 0.605 | 0.803 | | | 0.058 | 0.073 | |
| **Follow-up time (years)** | | | | | | | | | | | | | | | | | | |
| 5 – 9 | 26/1.9 | 13.5 (8.8, 19.7) | 46.1 (27.0, 65.3) | 22/0.2 | 141.5 (88.7, 214.2) | 41.9 (24.2, 59.5) | 90.9% | 19/0.0 | 36.4 (20.0, 52.8) | 79.0% | NR | 19.3 (4.0, 56.4) | 5.5 (-1.1, 12.0) | 11.9% | NR | 2.3 (0.6, 5.8) | 4.3 (-3.2, 11.8) | 9.3% |

| | All causes | | | Neoplastic causes | | | | Recurrence/progression | | | SPN | | | | Non-neoplastic causes | | | |
|---|---|---|---|---|---|---|---|---|---|---|---|---|---|---|---|---|---|---|
| **Characteristic** | **Obs/Exp** | **SMR (95% CI)** | **AER (95% CI)** | **Obs/Exp** | **SMR (95% CI)** | **AER (95% CI)** | **AER %** | **Obs/Exp** | **AER (95% CI)** | **AER %** | **Obs/Exp** | **SMR (95% CI)** | **AER (95% CI)** | **AER %** | **Obs/Exp** | **SMR (95% CI)** | **AER (95% CI)** | **AER %** |
| 10+ | 12/1.5 | 7.8 (4.0, 13.6) | 39.1 (13.7, 64.5) | 7/0.1 | 67.6 (27.2, 139.2) | 25.8 (6.4, 45.2) | 66.0% | NR | 15.0 (0.3, 29.6) | 38.4% | NR | 29.0 (6.0, 84.6) | 10.8 (-1.9, 23.5) | 27.6% | 5/1.4 | 3.5 (1.1, 8.1) | 13.3 (-3.1, 29.7) | 34.0% |
| *p* for trend | | 0.106 | 0.671 | | 0.072 | 0.252 | | | 0.077 | | | 0.620 | 0.425 | | | 0.516 | 0.282 | |
| **Residence zone at diagnosis** | | | | | | | | | | | | | | | | | | |
| South | NR | 14.4 (3.0, 42.0) | 57.7 (-12.5, 127.9) | NR | 119.0 (14.4, 429.8) | 41.0 (-16.3, 98.3) | 71.1% | NR | 20.7 (-19.8, 61.2) | 35.9% | NR | 59.5 (1.5, 331.5) | 20.3 (-20.2, 60.8) | 35.2% | NR | 5.2 (0.1, 29.0) | 16.7 (-23.8, 57.2) | 28.9% |
| Calgary | 10/1.1 | 9.1 (4.4, 16.8) | 34.3 (10.4, 58.1) | 8/0.1 | 95.1 (41.0, 187.3) | 30.5 (9.1, 51.8) | 88.9% | 5/0.0 | 19.2 (2.4, 36.1) | 56.0% | NR | 35.6 (7.4, 104.2) | 11.2 (-1.8, 24.3) | 32.7% | NR | 2.0 (0.2, 7.1) | 3.8 (-6.9, 14.5) | 11.1% |
| Central | 8/0.5 | 16.1 (6.9, 31.7) | 64.8 (16.9, 112.6) | 6/0.0 | 160.3 (58.8, 348.9) | 51.5 (10.0, 92.9) | 79.5% | 6/0.0 | 51.8 (10.4, 93.2) | 79.9% | 0 | - | - | 0.0% | NR | 4.3 (0.5, 15.7) | 13.3 (-10.6, 37.2) | 20.5% |
| Edmonton | 12/1.1 | 10.8 (5.6, 18.8) | 43.3 (16.3, 70.4) | 9/0.1 | 110.0 (50.3, 208.9) | 35.5 (12.1, 58.9) | 82.0% | 9/0.0 | 35.8 (12.4, 59.2) | 82.7% | 0 | - | - | 0.0% | NR | 2.9 (0.6, 8.5) | 7.8 (-5.7, 21.3) | 18.0% |
| North | 5/0.6 | 9.1 (2.9, 21.1) | 39.5 (0.6, 78.4) | NR | 103.5 (28.2, 265.0) | 35.2 (0.4, 70.0) | 89.1% | NR | 17.8 (-6.9, 42.4) | 45.1% | NR | 51.8 (6.3, 187.0) | 17.4 (-7.2, 42.0) | 44.1% | NR | 1.9 (0.0, 10.8) | 4.3 (-13.1, 21.7) | 10.9% |
| *p* for heterogeneity | | 0.773 | 0.794 | | 0.917 | 0.920 | | | 0.445 | | | 0.104 | 0.106 | | | 0.898 | 0.904 | |
| **Treatment Received** | | | | | | | | | | | | | | | | | | |
| Chemotherapy | 32/2.3 | 13.7 (9.4, 19.4) | 54.6 (34.2, 75.0) | 26/0.2 | 149.6 (97.7, 219.2) | 47.5 (29.1, 65.9) | 87.0% | 20/0.0 | 36.8 (20.7, 53.0) | 67.4% | 6/0.2 | 34.5 (12.7, 75.1) | 10.7 (1.9, 19.6) | 19.6% | 6/2.2 | 2.8 (1.0, 6.0) | 7.1 (-1.8, 15.9) | 13.0% |
| No Chemotherapy | 6/1.1 | 5.3 (1.9, 11.5) | 19.8 (0.2, 39.3) | NR | 35.2 (7.3, 102.8) | 11.9 (-2.0, 25.7) | 60.1% | NR | 12.2 (-1.6, 26.0) | 61.6% | 0/0.1 | 0.0 (N/A, N/A) | N/A (N/A, N/A) | 0.0% | NR | 2.8 (0.6, 8.3) | 7.9 (-5.9, 21.7) | 39.9% |
| *p* for heterogeneity | | 0.017 | 0.028 | | 0.004 | 0.008 | | | 0.042 | | | 0.029 | 0.038 | | | 0.972 | 0.918 | |
| Surgery | 11/1.6 | 6.9 (3.4, 12.4) | 25.9 (8.0, 43.8) | 9/0.1 | 75.1 (34.3, 142.5) | 24.4 (8.3, 40.6) | 94.2% | 8/0.0 | 22.0 (6.8, 37.3) | 84.9% | 1/0.1 | 8.3 (0.2, 46.5) | 2.4 (-3.0, 7.8) | 9.3% | NR | 1.4 (0.2, 4.9) | 1.5 (-6.2, 9.1) | 5.8% |
| No Surgery | 27/1.9 | 14.4 (9.5, 20.9) | 59.0 (35.1, 83.0) | 20/0.1 | 143.7 (87.8, 221.9) | 46.7 (26.1, 67.3) | 79.2% | 15/0.0 | 35.3 (17.4, 53.1) | 59.8% | 5/0.1 | 35.9 (11.7, 83.8) | 11.4 (1.1, 21.7) | 19.3% | 7/1.7 | 4.0 (1.6, 8.3) | 12.4 (0.2, 24.5) | 21.0% |
| *p* for heterogeneity | | 0.033 | 0.031 | | 0.094 | 0.099 | | | 0.272 | | | 0.125 | 0.128 | | | 0.141 | 0.138 | |

| | All causes | | | Neoplastic causes | | | | Recurrence/progression | | | SPN | | | | Non-neoplastic causes | | | |
|---|---|---|---|---|---|---|---|---|---|---|---|---|---|---|---|---|---|---|
| **Characteristic** | **Obs/Exp** | **SMR (95% CI)** | **AER (95% CI)** | **Obs/Exp** | **SMR (95% CI)** | **AER (95% CI)** | **AER %** | **Obs/Exp** | **AER (95% CI)** | **AER %** | **Obs/Exp** | **SMR (95% CI)** | **AER (95% CI)** | **AER %** | **Obs/Exp** | **SMR (95% CI)** | **AER (95% CI)** | **AER %** |
| Radiotherapy | 29/1.2 | 23.4 (15.7, 33.6) | 112.4 (69.7, 155.1) | 25/0.1 | 279.2 (180.7, 412.2) | 100.8 (61.2, 140.5) | 89.7% | 20/0.0 | 81.0 (45.5, 116.5) | 72.1% | 5/0.1 | 55.8 (18.1, 130.3) | 19.9 (2.1, 37.6) | 17.7% | NR | 3.5 (0.9, 8.9) | 11.5 (-4.3, 27.4) | 10.2% |
| No radiotherapy | 9/2.2 | 4.0 (1.8, 7.7) | 12.5 (1.6, 23.3) | NR | 23.6 (6.4, 60.4) | 7.1 (-0.2, 14.3) | 56.8% | NR | 5.5 (-0.7, 11.8) | 44.0% | NR | 5.9 (0.1, 32.9) | 1.5 (-2.1, 5.1) | 12.0% | 5/2.1 | 2.4 (0.8, 5.7) | 5.4 (-2.7, 13.5) | 43.2% |
| *p* for heterogeneity | | <0.001 | <0.001 | | <0.001 | <0.001 | | | <0.001 | | | 0.014 | 0.009 | | | 0.594 | 0.472 | |
| Transplant | 10/0.3 | 38.4 (18.4, 70.7) | 188.4 (68.5, 308.4) | 9/0.0 | 499.3 (228.3, 947.8) | 173.8 (60.0, 287.5) | 92.3% | 8/0.0 | 154.8 (47.5, 262.0) | 82.2% | NR | 55.5 (1.4, 309.1) | 19.0 (-18.9, 56.9) | 10.1% | NR | 4.1 (0.1, 23.0) | 14.7 (-23.3, 52.6) | 7.8% |
| No transplant | 28/3.2 | 8.7 (5.8, 12.6) | 33.6 (19.6, 47.7) | 20/0.2 | 83.0 (50.7, 128.1) | 26.8 (14.9, 38.7) | 79.8% | 15/0.0 | 20.3 (10.0, 30.6) | 60.4% | 5/0.2 | 20.7 (6.7, 48.4) | 6.5 (0.5, 12.4) | 0.0% | 8/3.0 | 2.7 (1.2, 5.3) | 6.8 (-0.7, 14.3) | 20.2% |
| *p* for heterogeneity | | <0.001 | <0.001 | | <0.001 | <0.001 | | | <0.001 | | | 0.422 | 0.397 | | | 0.703 | 0.645 | |

Obs, observed; Exp, expected; SMR, standardized mortality ratio; AER, absolute excess risk; SPN, subsequent primary neoplasm; CNS, central nervous system; *p*, *p*-value; NR, not reportable (i.e. observed count <5).
